# Supplementary figures and images for: Survival Outcomes of Patients with Mantle Cell Lymphoma: A Retrospective, 15-Year, Real-Life Study
Source: Hematol Rep. 2024 Jan 18;16(1):50–62. doi: 10.3390/hematolrep16010006 (PMC10801596; doi:10.3390/hematolrep16010006)

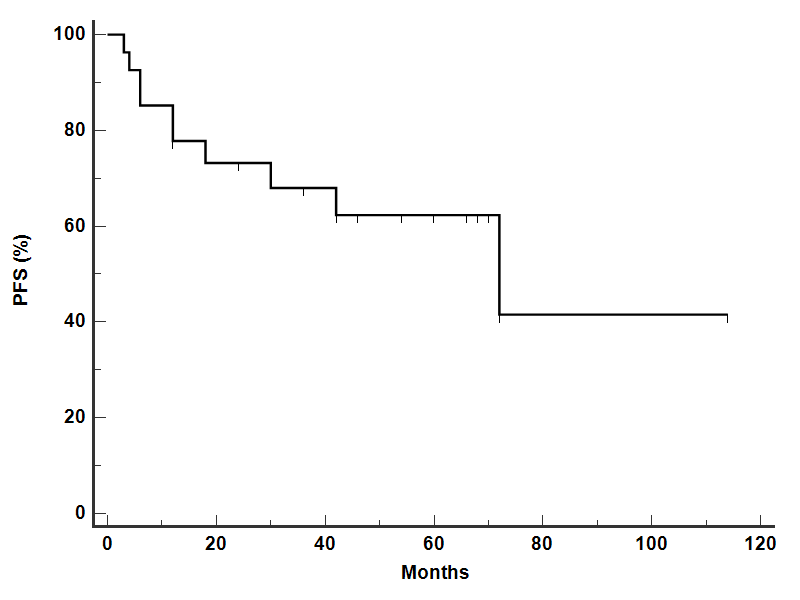

Supplement: Supplementary file 1 [file hematolrep-16-00006-s001.zip › Fig. S1 PFS less than 65.tif]

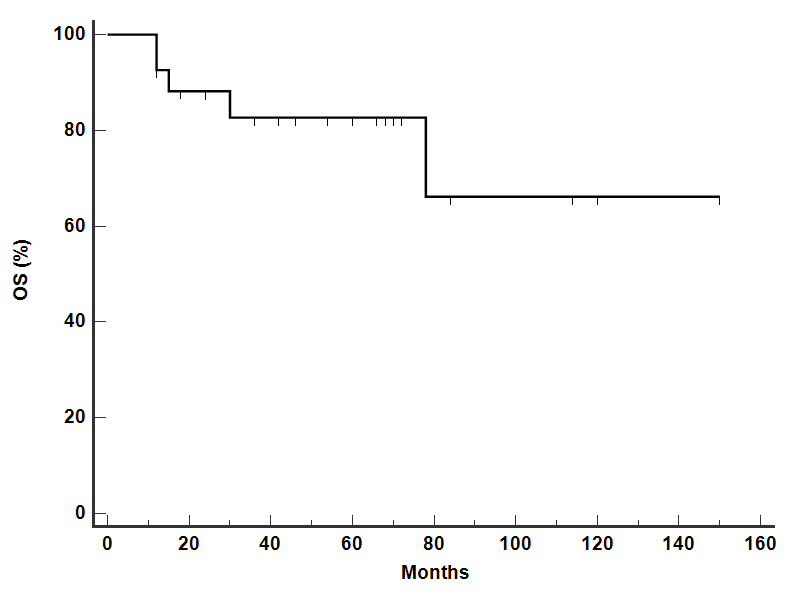

Supplement: Supplementary file 1 [file hematolrep-16-00006-s001.zip › Fig. S2 OS less than 65.tif]

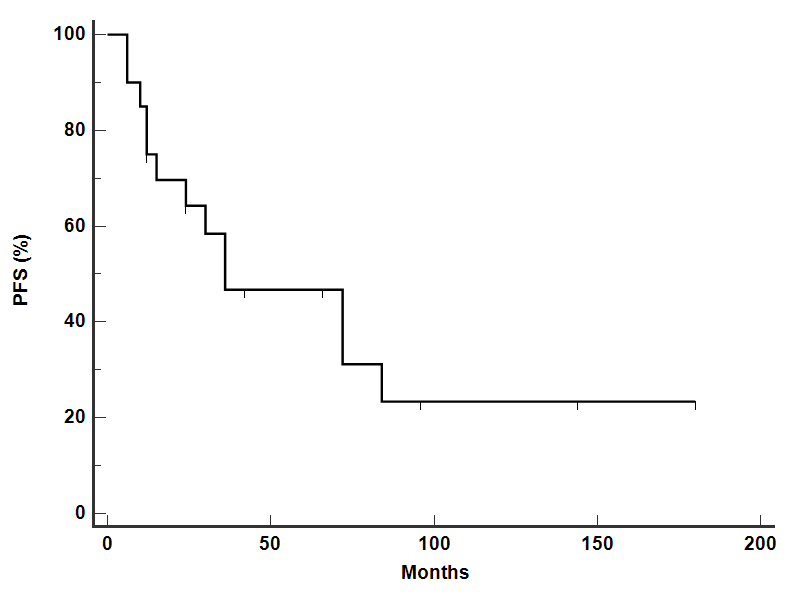

Supplement: Supplementary file 1 [file hematolrep-16-00006-s001.zip › Fig. S3 PFS 65-74y.tif]

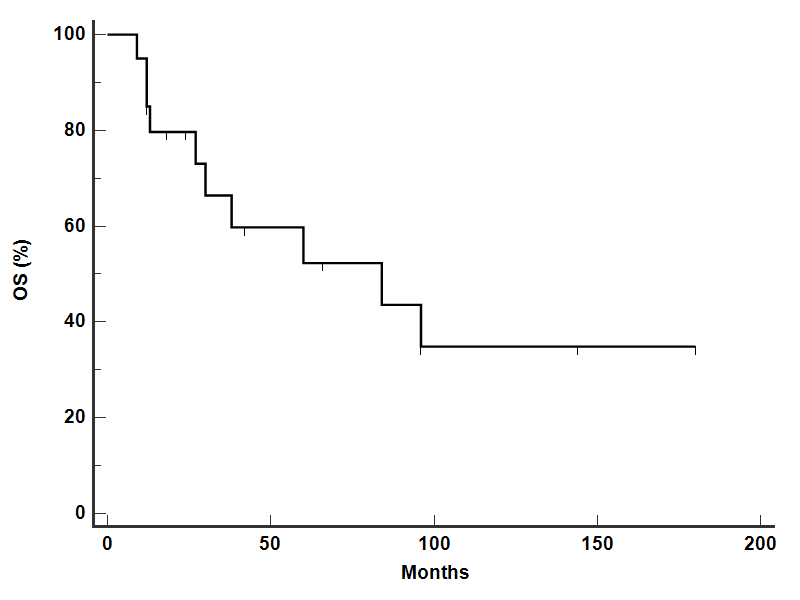

Supplement: Supplementary file 1 [file hematolrep-16-00006-s001.zip › Fig. S4 OS 65-74y.tif]

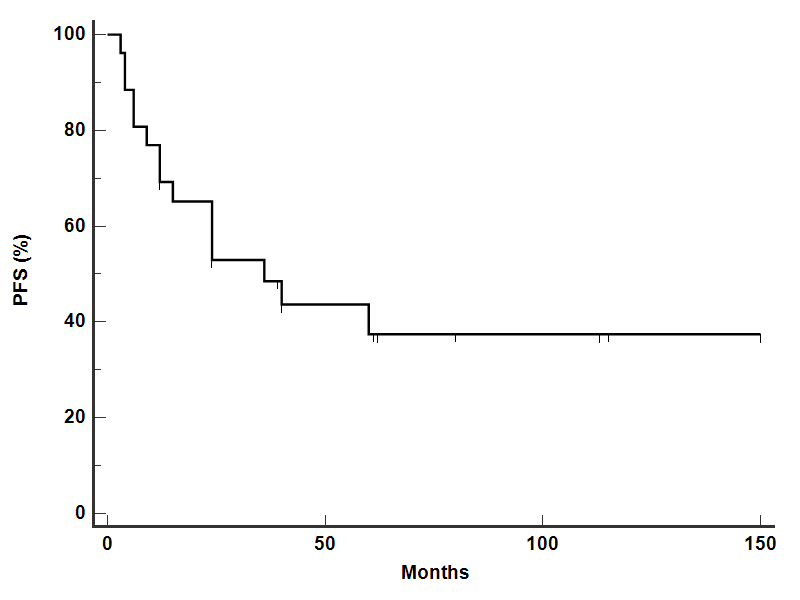

Supplement: Supplementary file 1 [file hematolrep-16-00006-s001.zip › Fig. S5 PFS more than 75y.tif]

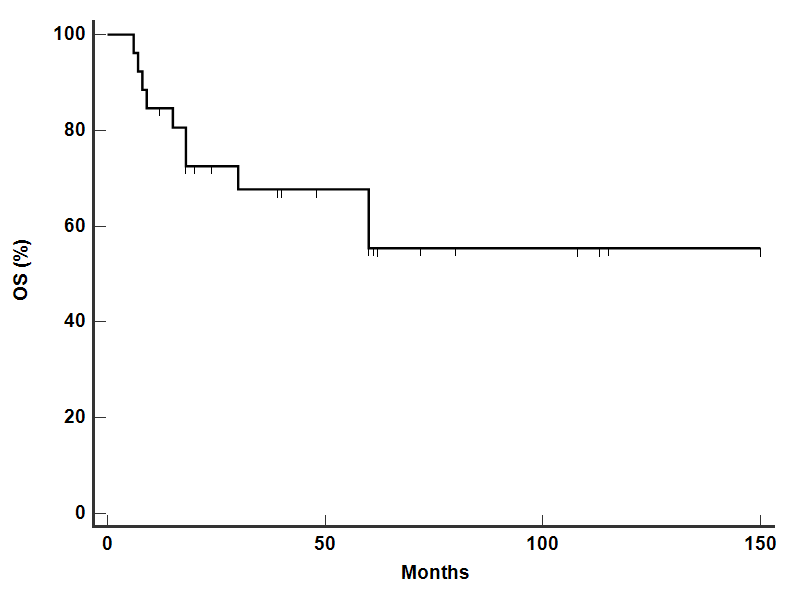

Supplement: Supplementary file 1 [file hematolrep-16-00006-s001.zip › Fig. S6 OS more than 75y.tif]
